# Isolation and phylogenetic characterization of Ebola viruses causing different outbreaks in Gabon.

**DOI:** 10.3201/eid0301.970107

**Published:** 1997

**Authors:** M C Georges-Courbot, A Sanchez, C Y Lu, S Baize, E Leroy, J Lansout-Soukate, C Tévi-Bénissan, A J Georges, S G Trappier, S R Zaki, R Swanepoel, P A Leman, P E Rollin, C J Peters, S T Nichol, T G Ksiazek

**Affiliations:** Centre International de Recherches Médicales, Franceville, Gabon.

## Abstract

Three outbreaks of Ebola hemorrhagic fever have recently occurred in Gabon. Virus has been isolated from clinical materials from all three outbreaks, and nucleotide sequence analysis of the glycoprotein gene of the isolates and virus present in clinical samples has been carried out. These data indicate that each of the three outbreaks should be considered an independent emergence of a different Ebola virus of the Zaire subtype. As in earlier Ebola virus outbreaks, no genetic variability was detected between virus samples taken during an individual outbreak.


### Isolation and Phylogenetic Characterization of Ebola Viruses Causing Different Outbreaks in Gabon

Three outbreaks of Ebola hemorrhagic fever have recently occurred in Gabon. Virus has been isolated from clinical materials from all three outbreaks, and nucleotide sequence analysis of the glycoprotein gene of the isolates and virus present in clinical samples has been carried out. These data indicate that each of the three outbreaks should be considered an independent emergence of a different Ebola virus of the Zaire subtype. As in earlier Ebola virus outbreaks, no genetic variability was detected between virus samples taken during an individual outbreak.

Since the first recognized outbreaks of human Ebola virus hemorrhagic fever in Africa in the 1970s, biological and genetic differences have been described among the four distinct Ebola viruses isolated: Zaire, Sudan, and Côte d'Ivoire, all isolated from humans, and Reston virus isolated from macaque monkeys from the Philippines (1-5).

Serologic evidence has suggested the presence of Ebola virus in Gabon since 1982 (6). Since late 1994, three apparently independent outbreaks of Ebola virus hemorrhagic fever have occurred among humans in northeastern Gabon, in the forested areas of equatorial Ogooué-Ivindo province. The first, which started in December 1994 in gold-panner encampments of far northeastern Gabon, in the Minkouka area (between 1°24' and 1°44' North and 12°49' and 12°59' East, Figure 1) near the Nouna River, had several laboratory-confirmed cases (7). The second, which began in early February 1996 in Mayibout village (1°07' North, 13°06' East, Figure 1) on the Ivindo River, resulted in 37 Ebola hemorrhagic fever cases (8). The only means of transportation between these two areas is by boat; Makokou, the closest town to them (0°33' North, 12°50' East, Figure 1), has the provincial hospital to which patients and contacts were transferred. The third outbreak, which is ongoing, started in July 1996 in the village of Booué (0°06' South, 11°57' East, Figure 1), where most of the cases occurred; however, scattered cases have been diagnosed in surrounding villages and towns. Some patients have even been transported to Libreville, probably during the incubation period of the disease. One patient was treated in South Africa, where a fatal nosocomial infection was subsequently reported in a health care worker (9,10); 43 deaths due to Ebola hemorrhagic fever have already been reported during this prolonged outbreak (11).

We report here the Ebola virus glycoprotein (GP) gene sequences obtained from human samples collected during each of the three Gabonese epidemics. The Gab280 sequence comes from blood collected 1 day before the death of a patient, from the Nouna area, during the 1994 outbreak. The Gab282 and Gab283 sequences were derived from blood collected during the spring 1996

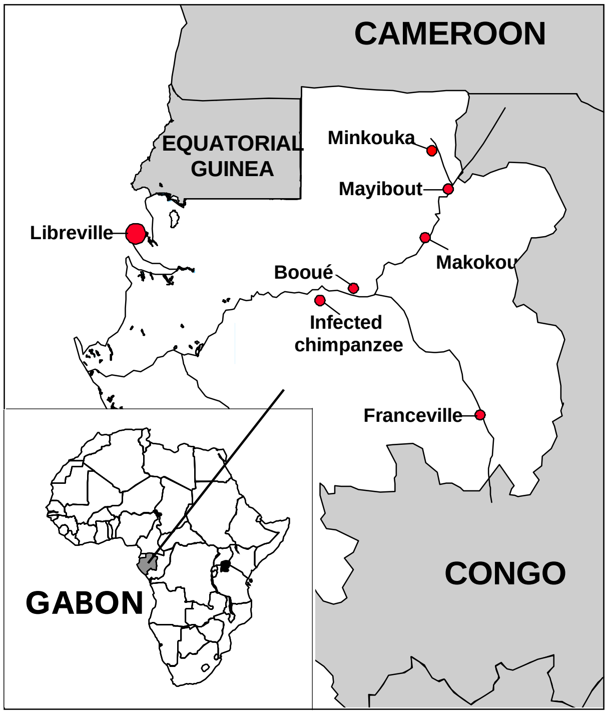

Figure 1. Geographic distribution of the three Ebola virus hemorrhagic fever epidemics and site of the infected chimpanzee in Gabon.

outbreak, from two primary patients who were infected while butchering a chimpanzee they found dead in the forest. The Gab281 sequence was derived from blood collected from what appears to have been a secondary case during the same outbreak; the patient was probably infected by contact with one of the index patients while visiting a traditional doctor (nganga) who lived near Mayibout village. Sequences Gab275 to Gab279 were derived from blood specimens collected from two patients during the fall 1996 outbreak: Gab275, Gab276, and Gab277 sequences are from one patient (a serum specimen collected on October 27, a primary tissue culture passage from that specimen, and a primary tissue culture passage from another blood sample drawn on October 25, respectively). The Gab278 and Gab279 sequences were derived from plasma (collected during the acute phase of illness) from another patient and its primary tissue culture passage, respectively. The isolation of Ebola virus in Vero E6 cell culture from human blood samples collected during the three different outbreaks was easily accomplished in a single passage.

RNA was extracted from blood or primary tissue culture samples (Table 1) by using a commercial kit (RNaid Kit , BIO 101, Inc., Vista, CA) as directed by the instruction manual. Viral sequences were amplified from RNA by using the reverse transcriptase-polymerase chain reaction (RT-PCR) technique, and DNA products were sequenced as described elsewhere (5). Briefly, amplified products were subjected to agarose electrophoresis and were stained and visualized with ethidium bromide; DNA bands were then excised and extracted by using the QIAEX II kit (QIAGEN, Inc., Chatsworth, CA). In some cases, nested PCR with internal primers was performed, using first-round products. Amplified products were directly sequenced by using an automated nonisotopic method (ABI PRISM dye-terminator cycle sequencing kit, Perkin-Elmer, Foster City, CA). Centri Sep columns (Princeton Separation, Inc.) were used to remove excess dye-labeled dideoxynucleotide terminators, and reaction products were analyzed on an ABI 377 or 373 DNA sequencer (Perkin-Elmer).

Initially, a 783-base-pair fragment of the GP gene, which encompasses the most variable region of the gene, was sequenced. A consensus sequence was established by aligning all the Ebola from Gabon, the Zaire 1976 and 1995 Ebola virus sequences, as well as the sequence of the virus obtained from a nurse in South Africa who was infected by a patient from Gabon in November 1996 (10). Viruses from the same outbreak had identical sequences, but unique virus sequences were found in each of the three different outbreaks in Gabon. Although the viruses causing the Gabonese outbreaks clearly belong to the Zaire subtype, they were distinct from viruses that had caused disease in Zaire. No differences were observed between tissue-culture-passaged and clinical-material-derived sequences or between primary or secondary case sequences. RNA extracted from a single representative of each outbreak (sequences Gab277, Gab280, and Gab283) was then used to generate the entire GP gene sequence for the Gabon Ebola viruses. The GP from the Gabon spring 1996 viruses differed from the sequence of the Gabon fall 1994 viruses by four nucleotides. The GP sequence from the Gabon fall 1996 viruses differed from the sequence of the Gabon spring 1996 virus by four additional nucleotides (Figure 2).

Maximum parsimony analyses were performed by using the entire coding regions of the GP genes of the Gabon viruses, together with those of

Table 1. Origin and identification of the sequence used in the analysis

| Outbreak | Patient # | Specimen | Date of original specimen | Laboratory # |
|---|---|---|---|---|
| Gabon, Fall 94 | 1 | Blood | Dec 28,94 | Gab280 |
| Gabon, Spring 96 | 1 | Blood | Feb 20,96 | Gab281 |
| | 2 | Blood | Feb 8,96 | Gab282 |
| | 3 | Blood | Feb 8,96 | Gab283 |
| Gabon, Fall 96 | 1 | Serum | Oct 27,96 | Gab275 |
| | | Vero E6 Passage 1 | Oct 27,96 | Gab276 |
| | | Vero E6 Passage 1 | Oct 25,96 | Gab277 |
| | 2 | Plasma | Oct 27,96 | Gab278 |
| | | Vero E6 Passage 1 | Oct 27,96 | Gab279 |
| South Africa, Fall 96 | 1 | Vero E6 Passage 1 | Nov 9,96 | SA253 |

previously characterized Ebola viruses (5). In addition, the sequence of a 1980 Marburg virus isolate (Musoke strain) was included to provide an outgroup in the analysis. The Phylogenetic Analysis Using Parsimony version 3.1.1 software, using the branch-and-bound search option (12), was run on a Power Macintosh 8100/110 (Apple Computer, Inc.). A 4:1 transversion:transition weighting was used in analyses. A single most parsimonious tree was obtained (Figure 2), and bootstrap analysis strongly supports a common evolutionary origin for the viruses associated with disease in Gabon and Zaire. Overall, these data indicate that the three Gabon outbreaks should be considered independent events, likely originating from different sources.

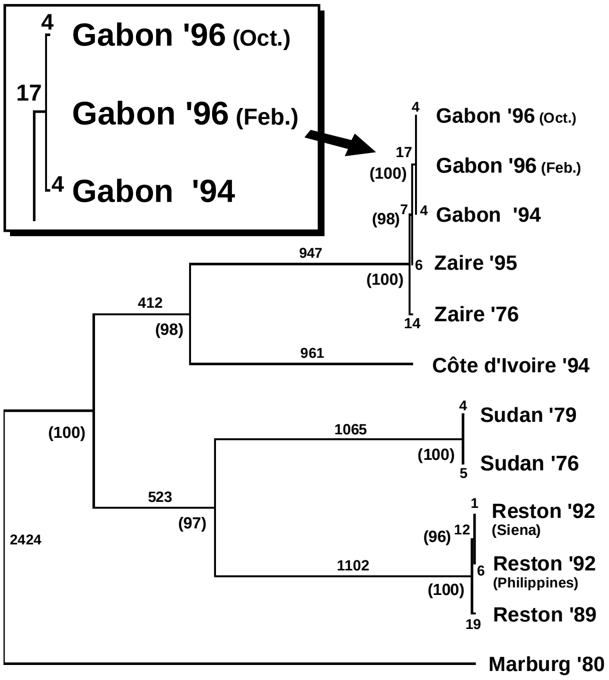

Figure 2. Phylogenetic tree showing the relationship between the Ebola viruses that caused outbreaks of disease in Gabon and previously described filoviruses (5). The entire coding region for the glycoprotein gene of the viruses shown was used in maximum parsimony analysis, and a single most parsimonious tree was obtained. Numbers in parentheses indicate bootstrap confidence values for branch points and were generated from 500 replicates (heuristic search). Branch length values are also shown.

The presence of stable virus sequences and the lack of genetic variability between strains isolated within an outbreak was previously seen during the outbreak of Ebola hemorrhagic fever in Kikwit, Zaire, in 1995 (Rodriguez et al., unpublished data), and despite the small number of isolates tested, is again suggested in Gabon.

During a 20-month period, Gabon has had three different outbreaks of Ebola virus hemorrhagic fever. The first and the second episodes apparently started during the rainy season (December and February), while the third began during the dry season (July). The deaths of nonhuman primates were associated with all three outbreaks. Minkouka area inhabitants reported finding dead chimpanzees and gorillas in the forest during the fall of 1994. All the primary human patients in the spring 1996 outbreak were infected while butchering dead chimpanzees. For the third outbreak, the investigation has indicated an index patient who was a hunter, living in a forest camp in the Booué area. During the same period, an Ebola virus-infected dead chimpanzee was found in the forest (0°11' South, 11°36' East) near Booué; Ebola virus infection in the chimpanzee was confirmed using a newly developed immunohistochemical Ebola skin biopsy test (Figure 3).

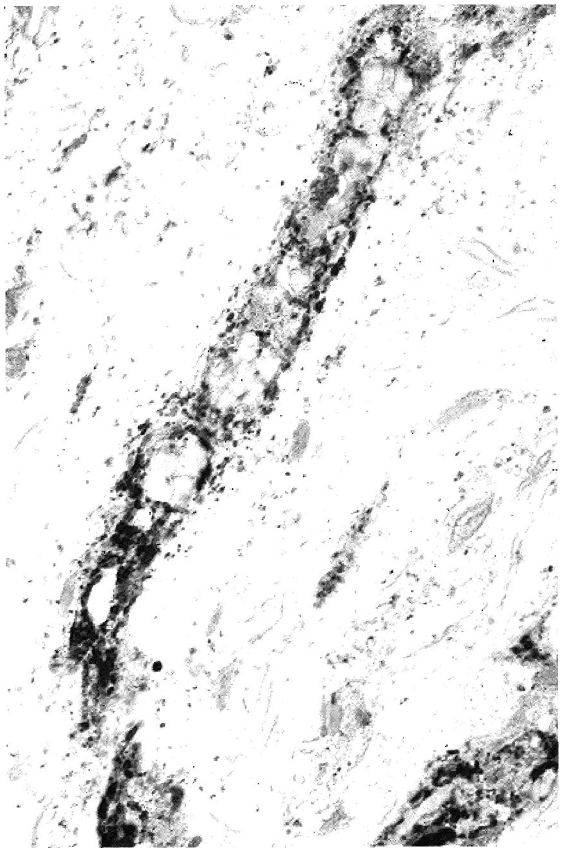

Figure 3. Immunostaining of Ebola virus antigens (red) within vascular endothelial cells in skin biopsy of the chimpanzee found dead in the forest near Booué. Note also the presence of extracellular viral antigens. (Rabbit anti-Ebola virus serum, napthol/fast red with hematoxylin counterstain, original magnification x250).

A long term surveillance for Ebola virus using this test is under way in Africa (13,14).

In Côte d'Ivoire in 1994, an investigator was infected with Ebola virus while performing necropsy on a dead chimpanzee (15). Primates are unlikely to be the reservoir of Ebola virus since experimental or natural infection is quickly fatal. A better knowledge of the ecology of great apes, particularly their food preferences and habitats, may lead to the identification of the virus reservoir. Gabon's equatorial forests, where three independent outbreaks have occurred in less than 2 years, offer an excellent opportunity for these investigations.

**Marie-Claude Georges-Courbot,* Anthony Sanchez,† Chang-Yong Lu,* Sylvain Baize,* Eric Leroy,* Joseph Lansout-Soukate,* Carole Tévi-Bénissan,* Alain J. Georges,* Sam G. Trappier,† Sherif R. Zaki,† Robert Swanepoel,‡ Patricia A. Leman,‡ Pierre E. Rollin,† Clarence J. Peters,† Stuart T. Nichol,† and Thomas G. Ksiazek†**

* Centre International de Recherches Médicales, BP 769 Franceville, Gabon;
†Centers for Disease Control and Prevention, Atlanta, Georgia, USA;
‡National Institute for Virology, Sandringham, South Africa

### References

1. Cox NJ, McCormick JB, Johnson KM, Kiley MP. Evidence for two subtypes of Ebola virus based on oligonucleotide mapping of RNA. J Infect Dis 1983; 147:272-5.
2. Fisher-Hoch SP, Brammer TL, Trappier SG, Hutwagner LC, Farrar BB, Ruo SL, et al. Pathogenic potential of filoviruses: role of geographic origin of primate host and virus strain. J Infect Dis 1992;166:753-63.
3. Jahrling PB, Geisbert TW, Jaax NK, Hanes MA, Ksiazek TG, Peters CJ. Experimental infection of cynomolgus macaques with Ebola-Reston filoviruses from the 1989-1990 US epizootic. Arch Virol 1996;11(Suppl):115-34.
4. Peters CJ, Sanchez A, Rollin PE, Ksiazek TG, Murphy F. Filoviridae: Marburg and Ebola viruses. In : Fields BN, Knipe DM, Howley PM, editors. Virology, 3rd ed., Philadelphia: Lippincott-Raven Publishers,1996:1161-76.
5. Sanchez A, Trappier SG, Mahy BWJ, Peters CJ, Nichol ST. The virion glycoproteins of Ebola viruses are encoded in two reading frames and are expressed through transcriptional editing. Proc Natl Acad Sci USA 1996;93:3602-7.
6. Ivanoff B, Duquesnoy P, Languillat G, Saluzzo J-F, Georges A, Gonzalez J-P, et al. Haemorrhagic fever in Gabon. I. Incidence of Lassa, Ebola and Marburg viruses in Haut-Ogooué. Trans R Soc Trop Med Hyg 1982;76:719-20.
7. Georges AJ, Renaut AA, Bertherat E, Baize S, Leroy E, LeGuenno B, et al. Recent Ebola virus outbreaks in Gabon from 1994 to 1996: epidemiologic and control issues. Proceedings of the International Colloquium on Ebola Virus Research; 1996 Sept 4-7; Antwerp, Belgium.
8. World Health Organization. Outbreak of Ebola haemorrhagic fever in Gabon officially declared over. Wkly Epidemiol Rec 1996;71:125-6.
9. World Health Organization. Ebola haemorrhagic fever—Gabon. Wkly Epidemiol Rec 1996;71:320.
10. World Health Organization. Ebola haemorrhagic fever—–South Africa. Wkly Epidemiol Rec 1996;71:359.
11. World Health Organization. Ebola haemorrhagic fever. Wkly Epidemiol Rec 1997;72:7.
12. Swofford DL, 1991. PAUP: phylogenetic analysis using parsimony, version 3.1.1. Champaign (IL): Illinois Natural History Survey.
13. Zaki SR, Greer PW, Goldsmith CS, Coffield LM, Rollin PE, Callain P, et al. Ebola virus hemorrhagic fever: pathologic, immunopathologic and ultrastructural studies. Proceedings of the International Colloquium on Ebola Virus Research; 1996 Sept 4-7; Antwerp, Belgium.
14. Lloyd E, Zaki SR, Rollin PE. Long term surveillance for Ebola in Zaire. Proceedings of the International Colloquium on Ebola Virus Research; 1996 Sept 4-7; Antwerp, Belgium.
15. LeGuenno B, Formenty P, Wyers M, Gounon P, Walker F, Boesch C. Isolation and partial characterization of a new strain of Ebola virus. Lancet 1995;345:1271-4.
